# Zebrafish Recoverin Isoforms Display Differences in Calcium Switch Mechanisms

**DOI:** 10.3389/fnmol.2018.00355

**Published:** 2018-09-28

**Authors:** Dana Elbers, Alexander Scholten, Karl-Wilhelm Koch

**Affiliations:** Department of Neuroscience, Biochemistry, University of Oldenburg, Oldenburg, Germany

**Keywords:** recoverin, photoreceptor, calcium-switch, conformational change, zebrafish

## Abstract

Primary steps in vertebrate vision occur in rod and cone cells of the retina and require precise molecular switches in excitation, recovery, and adaptation. In particular, recovery of the photoresponse and light adaptation processes are under control of neuronal Ca^2+^ sensor (NCS) proteins. Among them, the Ca^2+^ sensor recoverin undergoes a pronounced Ca^2+^-dependent conformational change, a prototypical so-called Ca^2+^-myristoyl switch, which allows selective targeting of G protein-coupled receptor kinase. Zebrafish (*Danio rerio*) has gained attention as a model organism in vision research. It expresses four different recoverin isoforms (zRec1a, zRec1b, zRec2a, and zRec2b) that are orthologs to the one known mammalian variant. The expression pattern of the four isoforms cover both rod and cone cells, but the differential distribution in cones points to versatile functions of recoverin in these cell types. Initial functional studies on zebrafish larvae indicate different Ca^2+^-sensitive working modes for zebrafish recoverins, but experimental evidence is lacking so far. The aims of the present study are (1) to measure specific Ca^2+^-sensing properties of the different recoverin isoforms, (2) to ask whether switch mechanisms triggered by Ca^2+^ resemble that one observed with mammalian recoverin, and (3) to investigate a possible impact of an attached myristoyl moiety. For addressing these questions, we employ fluorescence spectroscopy, surface plasmon resonance (SPR), dynamic light scattering, and equilibrium centrifugation. Exposure of hydrophobic amino acids, due to the myristoyl switch, differed among isoforms and depended also on the myristoylation state of the particular recoverin. Ca^2+^-induced rearrangement of the protein-water shell was for all variants less pronounced than for the bovine ortholog indicating either a modified Ca^2+^-myristoyl switch or no switch. Our results have implications for a step-by-step response of recoverin isoforms to changing intracellular Ca^2+^ during illumination.

## Introduction

Light-dependent changes in the second messenger concentration of cGMP and Ca^2+^-ions control the photoresponse characteristics of vertebrate rod and cone cells (Arshavsky and Burns, 2012; Koch and Dell’Orco, 2013). Feedback control of phototransduction in rod and cone cells crucially depends on cytoplasmic Ca^2+^ that is detected by photoreceptor specific neuronal Ca^2+^-sensor (NCS) proteins. Recoverin is a NCS protein and inhibits G protein-coupled receptor kinase GRK1, also known as rhodopsin kinase, at high levels of free Ca^2+^ concentration. Biochemical *in vitro* data and results on recoverin knockout mice support such a role of recoverin (Kawamura, 1993; Gorodovikova et al., 1994; Chen et al., 1995; Klenchin et al., 1995; Senin et al., 1995; Makino et al., 2004). However, several questions concerning the physiological role of recoverin are still under debate (Morshedian et al., 2018). Strongly coupled with recoverin’s function is its so-called Ca^2+^-myristoyl switch (Zozulya and Stryer, 1992; Dizhoor et al., 1993). Recoverin is posttranslationally modified with a myristoyl group at its N-terminus. In the Ca^2+^-free state, this acyl moiety is buried inside a hydrophobic cleft. Under saturating Ca^2+^ concentrations, the two functional Ca^2+^-binding sites in recoverin are occupied and the myristoyl group is exposed and can thereby interact with phospholipid membranes (Ames et al., 1995; Senin et al., 2002).

The pronounced conformational change and subsequent membrane translocation in response to changes in Ca^2+^ have made recoverin a working model for myristoyl switch mechanisms (Ames et al., 1997; Lange and Koch, 1997). Recent studies address the influence of lipid composition and membrane fluidity upon interaction of recoverin with membranes (Calvez et al., 2016; Potvin-Fournier et al., 2016, 2017; Yang et al., 2016), the impact on the structural organization of phospholipids (Brand and Koch, 2018), the orientation of membrane anchored recoverin (Timr et al., 2017), the intermediate states during Ca^2+^-dependent conformational transitions investigated by molecular dynamics simulations (Timr et al., 2018), and the complex interactions of recoverin with liposomes and CaF_2_ nanoparticles (Marino et al., 2014).

The zebrafish (*Danio rerio*) retina expresses four recoverin genes, *rcv1a*, *rcv1b*, *rcv2a*, and *rcv2b* coding for proteins zRec1a, zRec1b, zRec2a, and zRec2b, respectively (Zang et al., 2015). These recoverin isoforms differ in their expression pattern in the adult retina as zRec1a was only found in rods and UV cones, whereas all other zRec forms are present in all cone photoreceptors, but immunohistochemical staining of zRec2a is also seen in bipolar cells. The *in vivo* function of zRec proteins was studied by a morpholino-based targeted gene knockdown approach and normal and spectrum electroretinography (ERG). Target binding and regulation is suggested to occur with orthologs of mammalian GRK1 and GRK7 (Wada et al., 2006; genes annotated as *grk1a*, *grk1b*, *grk7a*, and *grk7b*) yielding pairs GRK1a-Rec1a (in rods) and GRK7a-Rec2a (in UV cones), but regulation of GRK1b and GRK7b by Rec2b is feasible as well (Rinner et al., 2005; Zang et al., 2015). According to the flash response data, zRec2a and zRec2b operate under different light regimes indicating different Ca^2+^-sensitive properties. Except for the studies of Zang et al. (2015), no molecular properties of zRec variants are known so far.

Guanylate cyclase-activating proteins (GCAPs) are related to recoverin and are activator/inhibitor proteins that regulate membrane bound sensory guanylate cyclases (Palczewski et al., 2004; Dizhoor et al., 2010; Koch and Dell’Orco, 2013). The variety of zRec isoforms is reminiscent of zebrafish GCAPs, of which six isoforms are expressed in the zebrafish retina (Imanishi et al., 2004; Rätscho et al., 2009). Detailed studies on zGCAP expression profiles in larval and adult animals, their regulatory properties, Ca^2+^ sensitivities, and conformational dynamics revealed a differential action mode for each protein supporting a Ca^2+^ relay mode of Ca^2+^-dependent negative feedback regulation (Scholten and Koch, 2011; Fries et al., 2012; Sulmann et al., 2015; Lim et al., 2017). We suggest a similar regulatory mode for zRec forms. A first step to unravel the mechanism of differential response properties of zRec to oscillating changes in cytoplasmic Ca^2+^ concentration is to investigate possible Ca^2+^-myristoyl switches, to determine their Ca^2+^-binding properties and conformational dynamics.

## Materials and Methods

### Protein Expression and Purification

All recoverin isoforms (zRec1a, zRec1b, zRec2a, and zRec2b) were heterologously expressed in *Escherichia coli* as described previously for bovine recoverin (bRec) (Lange and Koch, 1997; Senin et al., 2002). The zRec cDNA were provided by Prof. Dr. Stephan Neuhauss (University of Zurich, Switzerland) and cloned into plasmids pET21a(+) (zRec2b) or pET11d(+) (zRec1a, zRec1b, and zRec2a) by standard cloning techniques. To obtain myristoylated isoforms, *E. coli* cells were cotransformed with the plasmid pBB131 containing a gene for the yeast *N*-myristoyltransferase. After cell lysis, the isoforms were extracted from the insoluble fraction by homogenization in 6 M guanidine-hydrochloride and following refolding by dialysis against Tris buffer (20 mM Tris, 150 mM NaCl, 0.1 mM DTT, pH 8.0). The soluble and insoluble fractions of all recoverin forms were purified by a hydrophobic interaction chromatography except of zRec1b, which was purified by an ammonium sulfate precipitation followed by a size exclusion chromatography. After purification, recoverin containing fractions were combined and dialyzed against 50 mM ammonium hydrogen carbonate to remove residual EGTA that is present from the hydrophobic interaction chromatography step, followed by a buffer exchange against decalcified 50 mM ammonium hydrogen carbonate (purified recoverin samples are shown in **Supplementary Figure S1A**). Degree of myristoylation was determined by reversed phase high-performance liquid chromatography as previously described (Lange and Koch, 1997; Senin et al., 2002) yielding 89% (zRec1a), 63% (zRec1b), 96% (zRec2a), 73% (zRec2b), and 96% (bRec).

### Antibodies

Recombinant non-myristoylated zrecoverin isoforms were used as antigens by a company (Pineda, Berlin, Germany) for producing polyclonal antibodies in rabbits. The obtained sera were further purified with an affinity chromatography column. For this purpose, each recombinant non-myristoylated recoverin (zRec1a, zRec1b, zRec2a, and zRec2b) was immobilized on a CNBr-activated sepharose column and the corresponding serum was passed over the column to remove unspecific antibodies. Due to the elution, all antibodies were diluted 1:10. Anti-zRec2a crossreacted also with zRec2b; therefore, two purification steps were necessary. The first step was catching cross-reactive antibodies by immobilizing zRec2b to the column and collecting the non-bound fraction, which was passed over the zRec2a affinity column in the second step. The specificities of all antibodies were tested *via* western blot (**Supplementary Figure S1B**).

### Ca^2+^-Dependent Membrane Binding

An equilibrium centrifugation assay was performed to study the membrane binding of all recoverin isoforms in a Ca^2+^-dependent manner (Senin et al., 2002). For this assay, 1 mg/ml recoverin in HEPES buffer (10 mM HEPES/KOH, 150 mM NaCl, 20 mM MgCl_2_, 1 mM DTT, pH 7.4) were incubated with 2 mg/ml urea-washed rod outer segment (ROS) membranes and 2 mM CaCl_2_ or 2 mM EGTA. After 30 min of incubation (25°C, 700 rpm), the samples were centrifuged (30 min, 13,000 rpm), supernatants were discarded, and pellets were resuspended with 2 mM CaCl_2_ or 2 mM EGTA in HEPES buffer to remove unbound recoverin. After additional 30 min of centrifugation, supernatants were discarded and pellets were resolved in sample buffer. The samples were separated by SDS-PAGE, followed by a protein transfer to a nitrocellulose membrane by a semi-dry blotting using Towbin buffer (25 mM Tris, 192 μM glycine, 20% methanol p.a.). After the transfer, all blots were blocked with 1% milk powder (1 h, RT) in TBS-T (20 mM Tris, 154 mM NaCl, 0.05 % Tween-20). After blocking, incubation followed with primary specific antibodies against zRec isoforms (see below) and against bRec (rabbit anti-Rec k/2^+^; Lambrecht and Koch, 1992). Primary antibodies in 1% milk powder in TBS-T were incubated for 1 h at room temperature using the following dilutions: for zRec1b (1:20,000), zRec2b (1:40,000), and bRec (1:30,000). Anti-zRec1a and Anti-zRec2a antibodies were incubated over night at 4°C (dilution: zRec1a at 1:20,000, zRec2a at 1:10,000). Incubation with primary antibodies was followed by incubation with the secondary antibody that was a goat anti-rabbit IgG coupled to peroxidase (Dianova) for 1 h at room temperature. Dilution was for all zRecs 1:20,000, and for bRec 1:30,000. Every incubation step was followed by different washing steps with TBS and TBS-T. For visualization, membranes were incubated for 1 min in WesternBright reagents (Avansta) and then exposed to LucentBlue X-ray films (Advansta). Signal intensity was determined by a densitometric analysis of antibody reactive bands with an AlphaImager (Biozym). For each band, the integral density value (IDV) was determined by measuring the density in a certain area and subtracting the background of the blot.

### Determination of Ca^2+^-Binding Constants

A chelator assay was employed for the determination of macroscopic Ca^2+^-binding constants (Linse, 2002; Dell’Orco et al., 2010a) with the following modifications. Oregon Green^TM^ 488 BAPTA-5N (Invitrogen) was used instead of Dibromo-BAPTA as the competing chelator. It has a *K*_D_ value of 20 μM in MOPS buffer (10 mM MOPS, 100 mM KCl, pH 7.2) (Agronskaia et al., 2004), thus matching the Ca^2+^-binding constants for myristoylated and non-myristoylated bRec (17 μM for myristoylated bRec, 0.11 and 6.9 μM for non-myristoylated bRec, respectively; Ames et al., 1995). Using non-myristoylated bRec as internal standard, we validated the method for our purpose yielding a pattern of similar apparent *K*_D_ values (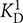 = 0.79 μM and 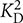 = 13.7 μM). Recoverin concentrations of stock solutions were determined by using a recoverin-specific Bradford calibration curve. Titrations were performed with 15 μM recoverin and 0.5 μM BAPTA-5N in decalcified MOPS buffer. To decalcify the buffer, the buffer passed several times over a Chelex column (Chelex 100^®^ sodium form, Sigma). The initial [Ca^2+^] in the titration was determined by BAPTA and ranged between 15 and 60 nM. Chelator fluorescence spectra were measured with a fluorescence spectrophotometer from Photon Technology International using excitation at 494 nm and recording emission spectra between 500 and 560 nm. The Ca^2+^ titration was performed by using calcium stocks of 3 mM and 30 mM CaCl_2_ in decalcified MOPS-buffer. In a total volume of 500 μl protein solution, 15 injections of 0.5 μl of 3 mM CaCl_2_, followed by seven injections of 0.5 μl of 30 mM CaCl_2_ were done. For each [Ca^2+^], the peak amplitude at 524 nm was determined, normalized, plotted as a function of free calcium concentration and fitted by the CaLigator software (Andre and Linse, 2002).

### Surface Plasmon Resonance

A Biacore 2000 and Biacore 3000 surface plasmon resonance (SPR) instrument (GE Healthcare) was used for detecting Ca^2+^-induced conformational changes of recoverin variants as described before for other NCS proteins (Dell’Orco et al., 2010b, 2012; Sulmann et al., 2014). Immobilization at high densities on a commercially available CM5 sensor chip (GE Healthcare) is a prerequisite for the detection of conformational transitions by SPR as previously outlined in detail (Sulmann et al., 2014). All myristoylated (myr) recoverin variants were immobilized by thiol coupling yielding sufficient immobilization densities between 4 and 13 ng × mm^-2^ (1000 RU correspond to 1 ng protein per mm^2^; myr bRec = 3.6-5.4 ng × mm^-2^, myr zRec1a = 7.9 ng × mm^-2^, myr zRec2a = 12.6-13.5 ng × mm^-2^, myr zRec2b = 11.1 ng × mm^-2^). After immobilization, increasing [Ca^2+^] in a decalcified Tris buffer (5 mM Tris, 100 mM KCl, pH 7.5) were injected and flushed over the protein surface. Control injections over an empty flow cell were performed and subtracted from the sample flow cell (Dell’Orco et al., 2010b, 2012; Sulmann et al., 2014). By plotting the amplitude of the RU signal as a function of free [Ca^2+^], the half maximal change of response amplitude was determined after normalization by SigmaPlot 13 with a dynamic curve fitting with sigmoidal equation of each experiment, followed by averaging all *K*_1/2_ values and determining the standard deviation. The experiment was only performed with myristoylated isoforms; it was not possible to immobilize non-myristoylated isoforms in a sufficient amount. Furthermore, immobilization and subsequent titrations also failed with zRec1b.

### Fluorescence Studies

Changes in fluorescence emission of 8-anilinonaphthalene-1-sulfonic acid (ANS) caused by interaction with exposed protein regions (Hughes et al., 1995; Gorczyca et al., 2003) were recorded with a fluorescence spectrophotometer from Photon Technology International. Lyophilized protein was dissolved in HEPES buffer (80 mM HEPES/KOH, 40 mM KCl, 1 mM DTT, pH 7.5) and protein concentration was determined by a Bradford assay (Bradford, 1976). For every experiment, 2 μM recoverin variant, 2.5 μM ANS, and a certain [Ca^2+^] in HEPES buffer were incubated for 20 min on ice. For every [Ca^2+^] above 1 μM, a CaCl_2_ stock solution in HEPES buffer was prepared, every [Ca^2+^] under 1 μM was adjusted by a mixture of K_2_H_2_EGTA and K_2_CaEGTA (Tsien and Pozzan, 1989). ANS fluorescence excitation was performed at 380 nm, and emission spectra were recorded between 400 and 550 nm. After every measurement, the cuvette was cleaned by 70% ethanol, 5 mM EGTA, 100% acetone, and several steps of water in between. Data recording and processing were done with the software Felix32 (Photon Technology International). Maximal fluorescence emission for every [Ca^2+^] was determined, normalized and plotted as a function of free [Ca^2+^]. In SigmaPlot 13, a dynamic curve fitting with sigmoidal equation was used to determine a half maximal value (*K*_1/2_) of each experiment, followed by averaging all *K*_1/2_ values and determining the standard deviation.

### Dynamic Light Scattering

Dynamic light scattering (DLS) measurements were performed with a Zetasizer Nano-S (Malvern Instruments). For the experiment a polystyrene, disposable, semi-micro cuvette (Ratiolab) was used. Refractive index and viscosity were set to 1.330 and 0.8872 cP (values for water), and temperature was set to 25°C with 2 min equilibration time. The measurement angle was 173° backscatter, and the analysis model was set to multiple narrow models (high resolution). For each measurement, a minimum of 11 runs with 30 repetitions were performed. The used Tris buffer (5 mM Tris, 100 mM KCl, pH 7.5) was filtered through a Rotilabo^®^syringe filter (Carl Roth, 0.22 μM PDVF). Lyophilized protein was dissolved in decalcified Tris-buffer, protein concentration was determined by a Bradford assay and adjusted to a final concentration of 10 μM. After adjusting either a calcium concentration or EGTA concentration of 1 mM, the protein solution was filtered by an Anotop^TM^ 10 filter (Whatman, 0.02 μm) and the measurement was started. After recording, mean and standard deviation for each recoverin variant was determined.

## Results

### Ca^2+^-Myristoyl Switch and Ca^2+^Affinity of zRec Variants

**FIGURE 1:**
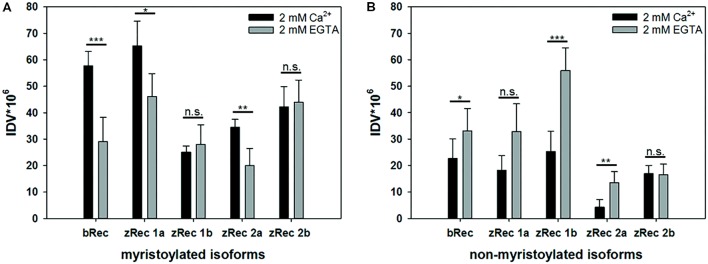
Ca^2+^-dependent membrane association of recoverin variants. An equilibrium centrifugation assay was performed with urea-washed ROS membranes and binding of myristoylated **(A)** and non-myristoylated **(B)** recoverin forms was probed in the presence (2 mM Ca^2+^) or absence (2 mM EGTA) of Ca^2+^. Variants of zRec and bRec were detected by isoform specific antibodies *via* western blotting, followed by a densitometric analysis of protein bands. IDV, integral density value; myr, myristoylated; nm, non-myristoylated. Shown is the mean ± s.d. *N* = 4, (EGTA) *N* = 5; nm zRec2a (Ca^2+^) *N* = 5, (EGTA) *N* = 4; myr zRec2b (Ca^2+^) *N* = 5, (EGTA) *N* = 7; nm zRec2b (Ca^2+^ and EGTA) *N* = 5.

Bovine recoverin shows a reversible binding to membranes triggered by changes in free Ca^2+^ concentration. This translocation process is connected to the functional role of mammalian recoverin controlling the activity of mammalian GRK1 in a Ca^2+^-dependent manner, inhibiting GRK1 at high Ca^2+^, and relieving the inhibition at low Ca^2+^. All zRec forms contain a consensus sequence for myristoylation and could in principle undergo a Ca^2+^-myristoyl switch and membrane translocation process. Therefore, we tested purified myristoylated and non-myristoylated zRec variants in a membrane binding assay using an equilibrium centrifugation assay. Surprisingly, only zRec1a, and to a lesser extent zRec2a, interacted with membranes in a Ca^2+^-dependent way like it is known from bRec (**Figure 1A**). bRec served as a benchmark control, since its Ca^2+^-dependent association with membranes or lipid mixtures is well described in test tube experiments that show 20-25% of total applied recoverin (Zozulya and Stryer, 1992; Senin et al., 2002). The other myristoylated zRec forms did not show a pronounced Ca^2+^ dependency during membrane binding. Instead, the non-myristoylated zRec forms exhibited an untypical binding pattern (**Figure 1B**) as all isoforms except zRec2b bound stronger to the membrane in their Ca^2+^-free state. By contrast, zRec2b showed no difference between its Ca^2+^-free and Ca^2+^-bound state.

**FIGURE 2:**
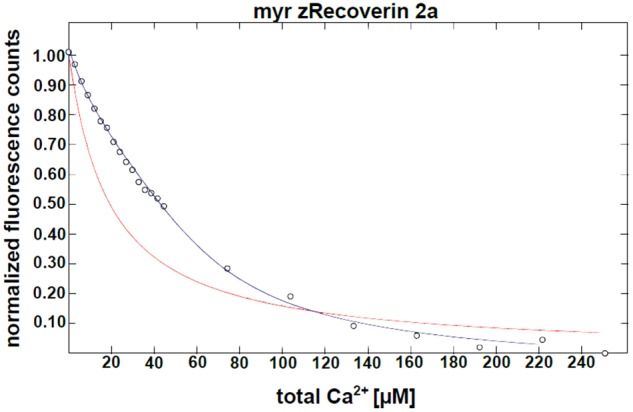
Ca^2+^ binding to myristoylated zRec2a by a competitive chelator assay. Chelator assay was performed with myristoylated (myr) zRec2a and Oregon Green^TM^ 488 BAPTA-5N. Chelator fluorescence spectra were measured by excitation at 494 nm and emission spectra were recorded between 500 and 560 nm. For each [Ca^2+^], the peak at 524 nm was normalized, plotted as a function of free calcium concentration and fitted by the CaLigator software. Titration with myristoylated zRec2a is a representative example of Ca^2+^ titration performed with all recoverin variants yielding an apparent 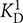 of 11.9 ± 2.3 μM. Red line corresponds to the titration of the chelator without protein, and the blue line corresponds to the titration in the presence of myristoylated zRec2a.

Furthermore, we determined the Ca^2+^-binding affinities of zRec forms by measuring macroscopic Ca^2+^-binding constants with a modified chelator method (**Figure 2** and **Table 1**). A representative example of a titration is displayed in **Figure 2** for myristoylated zRec2a revealing an apparent *K*_D_ of 11.9 μM (**Table 1**), which is close to the apparent *K*_D_ values of 14-17 μM obtained previously with bRec (Ames et al., 1995; Senin et al., 2002; Weiergräber et al., 2006). Myristoylated zRec1a with an apparent *K*_D_ of 15.4 μM fell also in this range. Larger differences became visible with myristoylated zRec1b and zRec2b revealing 9.2 and 23.4 μM, respectively (**Table 1**). Titrations with non-myristoylated zRec isoforms allowed the determination of two apparent *K*_D_ values. Fitting of binding curves gave for all zRec isoforms one apparent *K*_D_ of higher affinity and one of lower affinity (**Table 1**) resembling the Ca^2+^-binding studies with non-myristoylated bRec (Section “Materials and Methods”; Ames et al., 1995; Senin et al., 2002; Weiergräber et al., 2006). Previous ^45^Ca^2+^-binding studies assigned the *K*_D_ values of different affinities to the functional EF hands 2 and 3 in bRec (Senin et al., 2002). Although our data seem to reproduce the general pattern of high and low affinities, they indicate in all cases lower affinity of non-myristoylated zrecoverin variants for Ca^2+^.

**Table 1:** Ca^2+^-binding parameter of recoverin isoforms determined by a chelator assay.

Recoverin isoform	AppK_D_^1^ (μM)	AppK_D_^2^ (μM)	*N*
myr zRec 1a	15.4 ± 2.4	–	7
nm zRec 1a	0.6 ± 0.2	20.5 ± 8.5	6
myr zRec 1b	9.2 ± 2.7	–	8
nm zRec 1b	5.0 ± 1.8	12.3 ± 7.9	7
myr zRec 2a	11.9 ± 2.3	–	5
nm zRec 2a	1.2 ± 0.7	26.9 ± 15.9	8
myr zRec 2b	23.4 ± 6.7	–	8
nm zRec 2b	3.9 ± 0.6	21.8 ± 2.95	6

*Determination of apparent *K*_*D*_ values (*App*K_*D*_^*1*^ and *App*K_*D*_^*2*^; mean ± s.d.) by using the chromophoric chelator BAPTA-5N conjugated with the fluorescence dye Oregon Green 488 as described in Section “Materials and Methods” and in the legend of **Figure 2**. All curves were fitted with two-site binding model yielding two constants for non-myristoylated recoverin forms. For myristoylated recoverin variants, we calculated one macroscopic binding constant according to *K*_*D*_ = 10^-(*log*^*^*K*^*^*1+log*^*^*K*^*^*2)/2*^ as previously described for GCAP1 variants (Dell’Orco et al., 2010a) taking into account that myristoylated bRec shows one Ca^*2+*^ affinity constant in other Ca^*2+*^-binding assays*.

### Conformational Changes

Although all myristoylated zRec forms bound Ca^2+^ with moderate, but different affinity, they differed in their Ca^2+^-dependent membrane attachment. A lack of Ca^2+^-dependent attachment to the membrane could indicate that these zRec forms do not exhibit a large or distinct conformational change leading to the typical Ca^2+^-myristoyl switch. Therefore, we tested for conformational transitions in zRec variants by three different independent methods, SPR, ANS fluorescence spectroscopy, and dynamic light scattering (DLS), which allow the investigation of conformational changes from different perspectives.

#### Surface Plasmon Resonance

**FIGURE 3:**
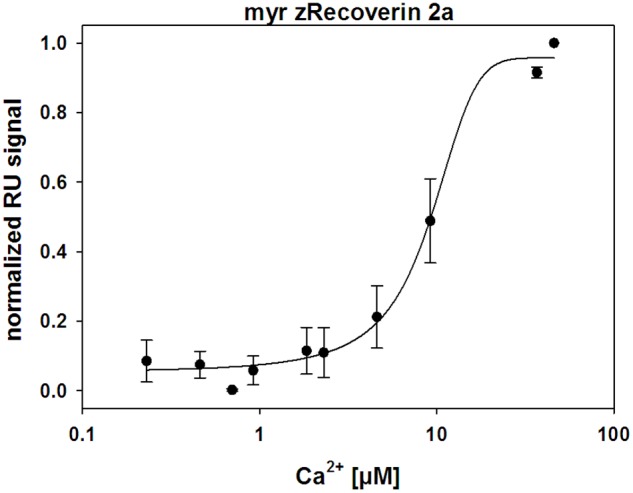
Ca^2+^-dependent conformational changes of immobilized zRec2a monitored by surface plasmon resonance. SPR response of zRec2a at increasing free [Ca^2+^]. Maximal amplitude of each [Ca^2+^] injection were normalized, averaged, and plotted as a function of free [Ca^2+^] and were fitted by SigmaPlot 13. Half maximal change of zRec2a occurred at 9.1 ± 1.7 μM. For statistical analysis and values obtained with other recoverin variants, see **Table 2**.

Ca^2+^-sensor proteins such as recoverin are well suited for monitoring conformational changes by SPR devices under precisely defined experimental conditions (Dell’Orco et al., 2010b, 2012; Sulmann et al., 2014). For this purpose, we immobilized zRec variants on hydrophilic dextran-coated sensor chips and injected increasing concentrations of CaCl_2_ in the nanomolar to micromolar range (**Figure 3**). All titrations with immobilized myristoylated zRec variants showed an increase in the maximal amplitudes at defined Ca^2+^ concentrations resembling those reversible changes in resonance units (RU) that were previously reported for bRec. **Table 2** gives a summary of the determined *K*_1/2_ values (Ca^2+^ concentration at which the increase in amplitudes is half-maximal). *K*_1/2_ values of zRec forms are around 9-10 μM and are thus slightly higher than 6.1 μM obtained for bRec and those reported in the literature (Dell’Orco et al., 2010b, 2012; Sulmann et al., 2014). These titrations demonstrate that zRec forms undergo distinct conformational changes reflecting changes in the hydrodynamic properties of the protein and that these changes seem to be triggered at similar [Ca^2+^]. However, they do not provide information about Ca^2+^-dependent exposition of domains or regions, about Ca^2+^-sensitive changes in hydrodynamic parameters or about the movement of the myristoyl group.

**Table 2:** Ca^2+^-triggered conformational changes in recoverin isoforms monitored by SPR.

Recoverin	*K*_1/2_ (μM)	Max. amplitude	RU/ng × mm^-2^	*N*
isoforms		(RU)		
myr bRec	6.1 ± 1.1	142.7 ± 1.5	39.6	16
myr zRec 1a	10.2 ± 0.8	50 ± 1.1	6.4	16
myr zRec 2a	9.1 ± 1.7	26 ± 1.4	1.9	32
myr zRec 2b	9.1 ± 1.4	43.5 ± 1.2	3.9	32

*Values of *K*_*1/2*_ are the free [Ca^*2*+^] at which SPR amplitudes are half maximal. Maximal amplitude in RU at saturating [Ca^*2+*^] and normalized per ng of immobilized protein; *N*, number of repetition (mean ± s.d.). Values for myristoylated zRec1b are missing due to difficulties of protein immobilization and long-term protein stability*.

#### ANS Fluorescence Spectroscopy

**FIGURE 4:**
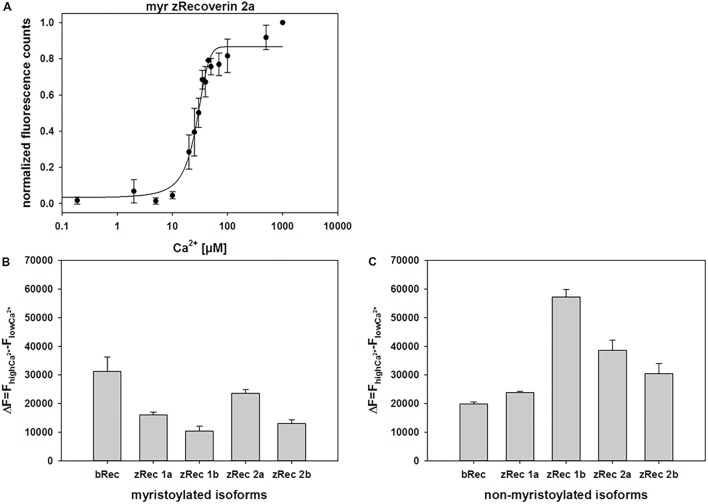
Normalized fluorescence emission of ANS interacting with myristoylated zRec2a. **(A)** Representative titration of 2 μM zRec2a with increasing [Ca^2+^] causing an increase of the ANS fluorescence emission. ANS fluorescence spectra were measured by excitation at 380 nm and emission spectra were recorded between 400 and 550 nm. Maximal fluorescence emission was normalized and plotted as a function of free [Ca^2+^]. After recording, all measurements of the same isoform were normalized, averaged and half maximal value (*K*_1/2_) was determined by SigmaPlot 13. *K*_1/2_ of myristoylated zRec2a was 22.6 ± 2 μM. **(B)** Fluorescence spectra of ANS were measured at high [Ca^2+^] of 500 μM and at low [Ca^2+^] of 1000 μM EGTA. Difference (Δ*F*) between high and low [Ca^2+^] showed the change in fluorescence intensity along myristoylated **(B)** and non-myristoylated **(C)** recoverin isoforms.

ANS interacts non-covalently with hydrophobic regions in proteins. Ca^2+^ binding to zRec variants could trigger the exposure or burying of hydrophobic parts (the myristoyl group or hydrophobic amino acid residues) and therefore, can monitor conformational changes of the whole protein by increasing (or decreasing) fluorescence emission. ANS fluorescence emission of myristoylated zRec2a was half-maximal at a *K*_1/2_ of 22.6 μM [Ca^2+^] (**Figure 4**) similar to the bovine control variant (24.6 μM, **Supplementary Table S1**). All other zRec forms showed significantly different *K*_1/2_ values being either fivefold lower (myristoylated zRec1b) or two- to-threefold higher (**Supplementary Table S1**). However, more unexpected differences became visible except for zRec1a, when we compared myristoylated and non-myristoylated variants (**Supplementary Table S1**) indicating a more specialized role of the myristoyl moiety for each zRec (see Section “Discussion” below).

We further analyzed difference fluorescence spectra providing information about the net fluorescence change between the Ca^2+^-saturating and the Ca^2+^-free state (**Figures 4B,C**). Most similar to the myristoylated bRec control was zRec2a, all other zRec variants exhibited lower net changes leading to the following sequence of Δ*F*: bRec > zRec2a > zRec1a > zRec2b > zRec1b. This pattern changed completely, when the myristoyl group was absent leading to a large net change in zRec1b, a lower change for other zRec forms that was still higher than the comparison with bRec (Δ*F*: zRec1b > zRec2a > zRec2b > zRec1a/bRec; **Figure 4C**). Maximal amplitudes of ANS fluorescence emission in the presence of Ca^2+^ gave an identical sequence (**Supplementary Table S2**).

#### Dynamic Light Scattering

**FIGURE 5:**
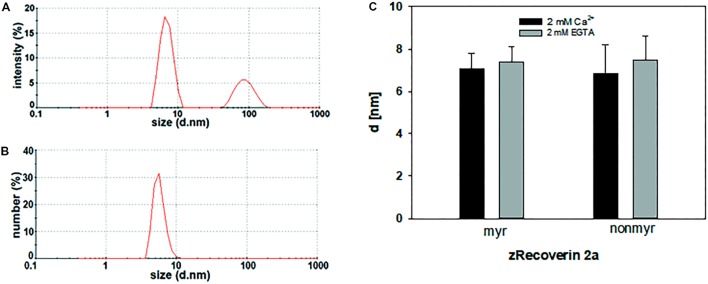
Dynamic light scattering measurement of zRec2a. Exemplary intensity **(A)** and number **(B)** plot of zRec2a in presence of Ca^2+^. DLS data reported the intensity of scattering light as % of the total area. The second peak in the intensity versus size plot originates from oligomeric or aggregated protein. However, the number plot refers to the relative percentage of sizes showing that only a very low percentage of the total protein is in oligomeric or aggregated form. **(C)** Comparison of the hydrodynamic diameter *d* (in nm) of zRec2a in the presence (2 mM Ca^2+^) and absence (2 mM EGTA) of Ca^2+^. For statistical analysis, see **Table 3**.

For dynamic light scattering, all recoverin isoforms were investigated in the absence and presence of Ca^2+^ to determine a potential difference in the conformation based on Ca^2+^ binding. Every variant was tested at least 40 times in a myristoylated and non-myristoylated form. To determine the hydrodynamic radius, an intensity plot of DLS data was used. The intensity plot showed one higher peak and one lower peak (**Figure 5A**). Concerning the absence of the second peak in a number plot (**Figure 5B**), the second peak was negligible and only the first peak was used for the further analysis. This peak reflected the size of the investigated protein revealing the hydrodynamic radius (**Figure 5C**). Myristoylated bRec showed the largest difference of the hydrodynamic radius between the Ca^2+^-bound and Ca^2+^-free state among all recoverin forms (**Table 3**) revealing for the Ca^2+^-bound state, a larger hydrodynamic radius of 7.54 ± 0.17 nm than for the Ca^2+^-free state, 6.15 ± 0.14 nm (**Table 3**). Nearly all other myristoylated zRec forms also showed a larger hydrodynamic radius in the Ca^2+^-bound state but the difference was not as prominent as for the bovine form. Only myristoylated zRec2a had a slightly larger hydrodynamic radius in the Ca^2+^-free state (**Table 3**).

**Table 3:** Dynamic light scattering measurements of recoverin isoforms.

Recoverin isoforms	d_Ca_ (nm)	*d*_EGTA_ (nm)	*N*	*N*	Δ*d* (nm)	Δ*d*/d_EGTA_ (%)	*P*-value d_Ca_/d_EGTA_
			d_Ca_	*d*_EGTA_			
myr bRec	7.54 ± 1.61	6.15 ± 1.29	90	85	1.39	22.6	<0.001
nm bRec	6.4 ± 0.76	6.34 ± 0.95	48	54	0.06	0.9	0.360
myr zRec 1a	6.79 ± 0.51	6.59 ± 1.25	54	44	0.2	3	0.305
nm zRec 1a	5.95 ± 0.71	6.59 ± 0.62	79	78	-0.65	-9.9	<0.001
myr zRec 1b	8.29 ± 1.75	8.01 ± 1.33	85	69	0.27	3.4	0.174
nm zRec 1b	6.57 ± 0.51	7.4 ± 1.62	54	59	-0.82	-11.1	0.01
myr zRec 2a	7.09 ± 0.70	7.4 ± 0.71	77	63	-0.329	-4.4	0.0037
nm zRec 2a	6.86 ± 1.33	7.5 ± 1.11	55	42	-0.64	-8.5	0.011
myr zRec 2b	5.82 ± 0.58	5.69 ± 0.59	54	55	0.13	2.3	0.145
nm zRec 2b	6.06 ± 0.52	6.07 ± 0.52	55	56	-0.02	-0.3	0.450

*Determination of the hydrodynamic diameter *d* ± s.d. in the presence (*d*_*Ca*_) and absence (*d*_*EGTA*_) of Ca^*2+*^. Nm, nanometer; *N*, number of repetition; Δ*d*, difference *d*_*Ca*_ -*d*_*EGTA*_. Statistical analysis was done by Student’s *t*-test (nm bRec, nm zRec1a, myr zRec2a, myr zRec2b, and myr zRec2b) or Mann–Whitney *U* test (myr bRec, myr zRec1a, myr zRec1b, nm zRec1b, and nm zRec2a). Recoverin isoforms with significant *P*-values of *d*_*Ca*_/*d*_*EGTA*_ are highlighted with a gray background; *P* ≥ 0.05, not significant.*

All non-myristoylated zRec forms except zRec2b have a larger hydrodynamic radius in the Ca^2+^-free than in the Ca^2+^-bound state (**Table 3**), which was opposite to non-myristoylated bRec, whose hydrodynamic radius was nearly the same in the Ca^2+^-bound (6.4 ± 0.11 nm) than in the Ca^2+^-free state (6.34 ± 0.13 nm).

## Discussion

Zebrafish recoverin isoforms share a high sequence homology to the bovine (or mammalian) ortholog (**Supplementary Figure S2**). However, our comparative analysis of the molecular properties of zebrafish recoverin forms demonstrate a pattern of significant differences, which we will discuss for each of the experimental approaches.

**FIGURE 6:**
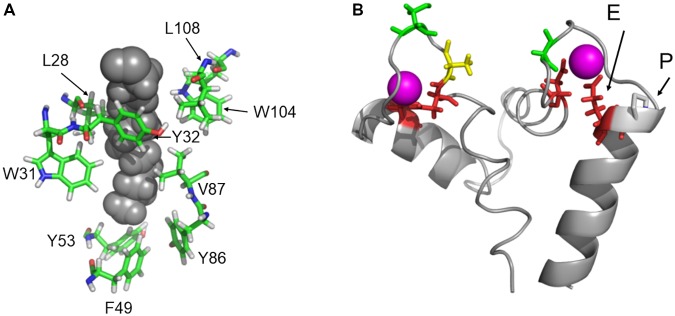
Protein regions that are important for recoverin function. **(A)** Highly conserved amino acid positions forming the hydrophobic myristoyl pocket. **(B)** EF hands 2 and 3 of bRec with Ca^2+^-bound (magenta). The essential Glu (E) is indicated in EF-hand 3. An Asn was replaced *in silico* by Pro (P) to illustrate a possible interference with the position of Glu. Images were created by Pymol using coordinates of Ca^2+^-free and Ca^2+^-bound bRec (PDB codes: 1IKU and 1JSA, Tanaka et al., 1995; Ames et al., 1997).

Mammalian recoverin, in particular bRec, is the prototype of those NCS proteins that undergo a Ca^2+^-myristoyl switch, which has been probed experimentally by measuring the Ca^2+^-dependent binding of myristoylated Rec forms to biological membranes (Zozulya and Stryer, 1992; Dizhoor et al., 1993; Senin et al., 2002). Other NCS proteins undergoing a Ca^2+^-myristoyl switch are, for example, hippocalcin, neurocalcin δ, and visinin-like proteins 1 and 3 (Kobayashi et al., 1993; Ladant, 1995; Spilker et al., 2000, 2002). In the Ca^2+^-free state, the myristoyl group is buried in a protein cleft consisting of hydrophobic amino acid side chains. All zRec variants have identical (or highly conserved) amino acids at the corresponding positions (**Figure 6A**) indicating that all Ca^2+^-free zRec isoforms keep the myristoyl group buried in a hydrophobic pocket. Critical residues for the Ca^2+^-triggered transition (and extrusion of the myristoyl group) are Gly42 and Gly96, around which rotational movements occur, and His68 and Leu108, which are involved in the changing domain interactions of EF-hand 2 and 3 (Ames et al., 2002). All these amino acids are identical in zRec variants and bRec. Differences in Ca^2+^-switch mechanisms as we see with zRec1b and zRec2b must therefore have other causes (see Section “Discussion” further below).

Unexpected was the higher binding of Ca^2+^-free non-myristoylated zRec1a, 1b, and 2a to membranes than binding of their Ca^2+^-saturated forms to membranes. Binding of proteins to membranes can be the result of hydrophobic and electrostatic interactions. Amino acid sequences of zRec isoforms differ in a few segments from those of bRec. Most of these short stretches of two to three amino acids show no consensus to corresponding amino acids in bRec. Furthermore, they are located on the protein surface (**Supplementary Figure S3**) and could account for the observed differences.

Ca^2+^ affinities of zRec isoforms should determine Ca^2+^sensitive properties such as the Ca^2+^-dependent membrane association. In mammalian and zebrafish recoverin forms, the two functional EF hands 2 and 3 are highly conserved in all critical positions with oxygen containing side chains (**Supplementary Figure S2**). However, in EF-hand 3 of zRec2b, a Pro is at the N-terminal part of the exiting helix and located next to the Glu that is essential for Ca^2+^ coordination. **Figure 6B** illustrates the position of Glu next to Pro that is substituted for Asn. Pro interfering with the helix structure could slightly disturb or shift the position of the complexing Glu leading to the lower Ca^2+^ affinity observed with zRec2b. Furthermore, the C-terminus in bRec was previously identified as an internal modulator of Ca^2+^ sensitivity. Truncation of C-terminal amino acids causes a shift to lower Ca^2+^ affinity in bRec (Weiergräber et al., 2006), which very likely determines the differences in Ca^2+^ affinity of zRec forms. In particular, not only zRec2b but also zRec2a lack the 12 or 10 amino acids, which are present in bRec. However, this C-terminal stretch is present in zRec1a and 1b with only some minor amino acid differences. Thus, the lack of the C-terminus cannot account alone for differences in Ca^2+^ sensitivities.

Binding of Ca^2+^ to bRec occurs in a sequential order with binding first to EF-hand 3 and second to EF-hand 2 (Permyakov et al., 2000; Senin et al., 2002), thereby triggering the conformational change. Binding of Ca^2+^ to zRec isoforms triggers in all variants conformational changes, which we could show by different experimental approaches. Changes in conformation detected by SPR spectroscopy was half maximal in the lower micromolar range in agreement with the Ca^2+^ affinity constants determined with the chelator assay, although differences in affinity constants are not directly mirrored by the SPR *K*_1/2_ values. We have observed and discussed this apparent mismatch in previous contributions concluding that the empirical parameter *K*_1/2_ reflects a concerted binding-conformational process (Dell’Orco et al., 2010b, 2012; Sulmann et al., 2014), which occur in different NCS proteins.

**FIGURE 7:**
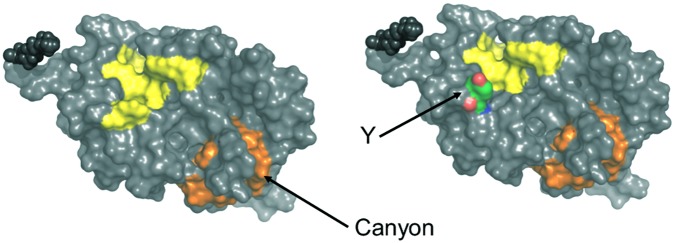
Surface presentation of myristoylated bRec with two bound Ca^2+^. Hydrophobic patches are indicated in yellow and orange. Replacement of Phe23 by Tyr (Y) is indicated on the right part of the image. Images were created by Pymol using coordinates of Ca^2+^-bound bRec (PDB code 1JSA).

ANS fluorescence emission is a tool to estimate the increase of solvent accessible hydrophobic surfaces depending on experimental conditions, for example, Ca^2+^-binding (Hughes et al., 1995; Gorczyca et al., 2003). It has previously been noticed that myristoylated and non-myristoylated bRec expose a large hydrophobic patch on Ca^2+^ binding (yellow labeled amino acids in **Figure 7**; Weiergräber et al., 2003). Aromatic and aliphatic amino acids constituting this hydrophobic patch are Leu28, Phe23, Trp31, Phe35, Ile44, Phe56, Phe57, Leu81, Phe83, and Leu90 and are identical in all zRec isoforms except for zRec1a and zRec1b, where Phe23 is replaced by Tyr. The increase in polarity by the presence of Tyr might decrease the general hydrophobicity of the patch (**Figure 7**) and could partially account for the lower Δ*F* value determined for zRec1a and even more for zRec1b, where the surface of the patch decreases.

Two other hydrophobic patches of lower size are visible on the surface of bRec, one made of Leu167, Phe172, Ile173, Leu187, and Ala188 and the second one made of Phe158, Phe159, Ile182, Leu183, Leu185, and Ile186. The latter one forms a kind of canyon-like structure, in which Ile182 in bRec is replaced by Ala and Gly in zRec2a and zRec2b, respectively (orange labeled, **Figure 7**). This hydrophobic groove might be another target of ANS. The Gly in zRec2b decreases the degree of hydrophobicity thereby lowering binding of ANS, whereas Ala in zRec2a has a side chain of lower hydrophobicity than Ile, but probably sufficient for ANS binding (**Supplementary Tables S1**, **S2**). In non-myristoylated bRec, the large hydrophobic patch is solvent accessible in both forms of bRec, with one or two Ca^2+^ bound (**Supplementary Figure S4** Weiergräber et al., 2003; Kumar et al., 2015). The hydrophobic canyon-like cleft seems to be exposed increasing the general hydrophobicity, although ANS binding to bRec occurred to nearly the same extent independent on the presence of the myristoyl group. Since we lack the three-dimensional structure of any zRec, we have no structure based explanation for the large increase in ANS fluorescence for the zRec isoforms, in particular for non-myristoylated zRec1b. We hypothesize that Ca^2+^-triggered conformational changes in zRec forms differ from those of bRec, which is already indicated by the differences in membrane association, but became even more apparent in our DLS data. All non-myristoylated zRec isoforms except zRec2b had a larger hydrodynamic radius in the Ca^2+^-free than in the Ca^2+^-bound state, which was opposite to bRec. Among all zRec forms, non-myristoylated zRec1b showed the largest difference, which correlates with the high Δ*F* value in **Figure 4C** and might even explain the unusual binding of zRec1b to membranes in the absence of Ca^2+^. A similar correlation of DLS data and the Δ*F* value was visible for non-myristoylated zRec2a, and this variant showed also higher binding to membranes in the absence of Ca^2+^ than in the presence, but to a general lower degree (**Figure 1B**). However, other zRec variants did not exhibit similar correlations.

We conclude from these observations that all zRec isoforms undergo Ca^2+^-triggered transitions, but some or all attain different conformations with consequences for the biochemical properties. Recent work by Zang et al. (2015) investigating recoverin deficient larvae suggested that zRec1a could be replaced by zRec2a. This is in agreement with both proteins sharing a similar Ca^2+^ sensitivity, which can be seen in their similar *K*_D_ values (**Table 1**). Zang et al. further showed that cone photoresponse recovery differs in zRec2a and zRec2b morphants depending on illumination that triggers transient changes in cytoplasmic Ca^2+^ concentration (Brockerhoff et al., 2003; Cilluffo et al., 2004; Leung et al., 2007). These findings are in agreement with the about twofold different Ca^2+^-binding constants that we determined for zRec2a and zRec2b (**Table 1**). Membrane binding and Ca^2+^-binding constants of zRec1a were the most similar to those of bRec. Due to the expression of zRec1a in rods (and UV cones) and the relationship of their targets zGRK1a and bovine GRK1, our results are in agreement with a functional pair of zRec1a and zGRK1a. Finally, we cannot exclude that one or more zRec forms target a different protein as this was observed for bRec interacting with caldendrin (Fries et al., 2010) and suggested for mammalian recoverin that is involved in the signal transmission between rod and bipolar cells (Sampath et al., 2005). For example, the high ability of non-myristoylated zRec forms to associate with membranes in low Ca^2+^ concentration in combination with the DLS data could point to conformational differences that are suitable for interacting with different targets.

## Author Contributions

DE, AS, and K-WK designed the study. DE and AS performed experiments. DE and K-WK analyzed data. K-WK wrote the first draft of the manuscript. All authors corrected and approved the final version of the manuscript.

## Conflict of Interest Statement

The authors declare that the research was conducted in the absence of any commercial or financial relationships that could be construed as a potential conflict of interest. The reviewer PG and handling editor declared their shared affiliation at the time of the review.
