# Supplementary material for: Zebrafish Recoverin Isoforms Display Differences in Calcium Switch Mechanisms
Source: Front Mol Neurosci. 2018 Sep 28;11:355. doi: 10.3389/fnmol.2018.00355 (PMC6172410; doi:10.3389/fnmol.2018.00355)
Supplement: Supplementary file 1 [file Table_1.DOCX]

**Supplementary information**

**Zebrafish recoverin isoforms display differences in calcium-switch mechanisms**

Dana Elbers^1^, Alexander Scholten^1^, Karl-Wilhelm Koch^1^

^1^Department of Neuroscience, Biochemistry, University Oldenburg, 26111 Oldenburg, Germany;

**Table S1:** Changes in ANS fluorescence emission interacting with recoverin isoforms as a function of free [Ca^2+^]. A Ca^2+^ titration was performed with myristoylated (myr) and non-myristoylated (nm) recoverin isoforms in the presence of the hydrophobic dye ANS. Plotting the normalized maximal fluorescence emission as a function of free [Ca^2+^] yielded a half maximal value (K_1/2_) of the titration (mean ± s.d.) , N = number of repetition.

| **Recoverin**  **isoforms** | **K_1/2_**  **[µM]** | **N** |
| --- | --- | --- |
| myr bRec | 24.6 ± 1.7 | 7 |
| nm bRec | 80.4 ± 4.1 | 4 |
| myr zRec 1a | 78.2 ± 9.9 | 6 |
| nm zRec 1a | 99.7 ± 21.5 | 4 |
| myr zRec 1b | 5.1 ± 0.6 | 6 |
| nm zRec 1b | 36.5 ± 2.4 | 4 |
| myr zRec 2a | 22.6 ± 2 | 6 |
| nm zRec 2a | 4.2 ± 0.9 | 8 |
| myr zRec 2b | 44.6 ± 8.7 | 5 |
| nm zRec 2b | 0.89 ± 0.03 | 8 |

**Table S2.** Maximal amplitudes of ANS fluorescence emission for all recoverin variants at saturating Ca^2+^ and in the absence of Ca^2+^ (1 mM EGTA). Order of maximal amplitudes (mean ± s.d.) was for myristoylated (myr) forms (+ Ca^2+^) bRec > zRec2a > zRec1a > zRec2b > zRec1b and for nonmyristoylated (nm) forms zRec1b > zRec2a > zRec2b > zRec1a ≈ bRec.

| **Recoverin**  **isoforms** | **Max. fluorescence intensity ANS 0.5 mM Ca^2+^** | **Max. fluorescence intensity ANS 1 mM EGTA** |
| --- | --- | --- |
| myr bRec | 37630.8 ± 5347.5 | 7649.4 ± 605.3 |
| nm bRec | 30606.2 ± 3117.6 | 11151.0 ± 2667 |
| myr zRec1a | 21260.1 ± 1422.9 | 6444.4 ± 1008.2 |
| nm zRec1a | 30968.9 ± 125.15 | 8296.6 ± 65.5 |
| myr zRec2a | 26151.7 ± 4522.9 | 5996.6 ± 891.5 |
| nm zRec 2a | 50166.9 ± 5026.2 | 12071.9 ± 1711.2 |
| myr zRec 1b | 15205.9 ± 2636.8 | 5641.2 ± 1248.5 |
| nm zRec 1b | 69043.7 ± 3523.1 | 11948.2 ± 1001.45 |
| myr zRec 2b | 17636.1 ± 1677.4 | 5672.2 ± 463.5 |
| nm zRec 2b | 35224.7 ± 5207.4 | 6287.9 ± 1255.4 |

**Figure S1**

**
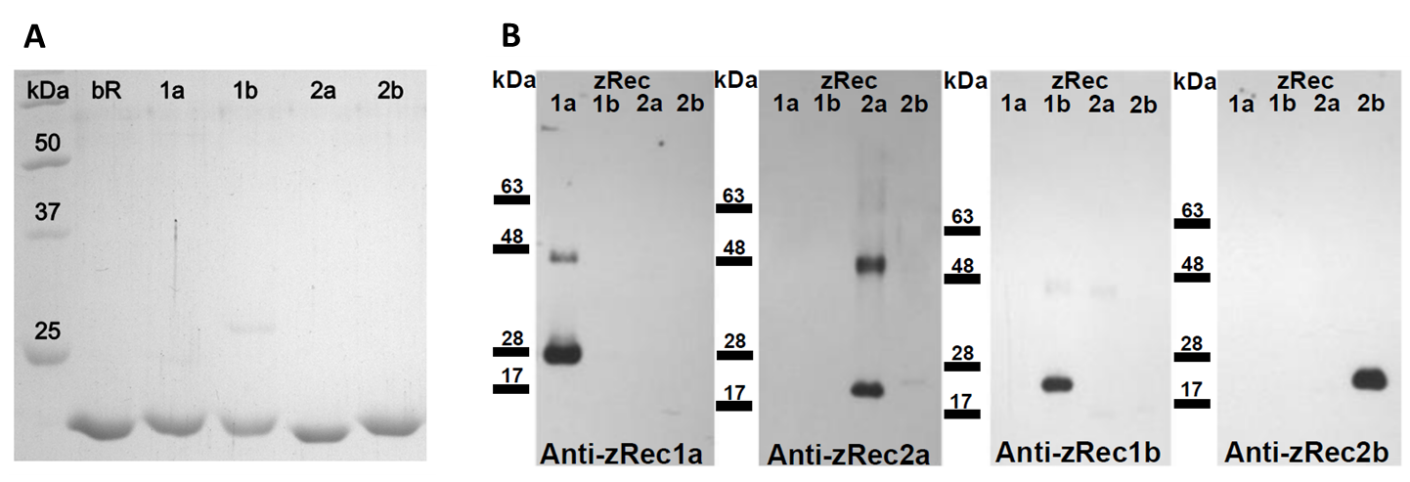
**

**Figure S1:** Test of antibody specificity. (A) SDS-polyacrylamide analysis of purified bovine recoverin (bR) and zRec isoforms. Gel was stained with Coomassie blue. (B) Purified zRec isoforms (10-25 ng in each lane) were analyzed by electrophoresis and immunoblotting using anti-zRec specific antibodies as indicated at the bottom of the image. Dilution of primary antibodies were: zRec1b (1:20,000), zRec2b (1:40,000). Anti-zRec1a and Anti-zRec2a antibodies were incubated over night at 4°C (dilution: zRec1a at 1:20,000, zRec2a at 1:10,000). Dilution for the secondary antibody was 1:20,000. zRec1a and zRec2a exhibited also dimeric forms. The gel corresponding to the immunoblot probed with anti-zRec1a had a shorter run time leading to a slightly different apparent electrophoretic mobility of zRec1a.

**Figure S2**

zRec1a MGNTKSGALSKELLEDLKLNTKYTEEELCAWYTSFLKECPSGRITKEQFEGIYASFFPDA

zRec1b MGNSRSSALSRDVLQELQTSTTYSQEQLFSWYQKFLNECPTGRISREQFQSIYASFFPDA

bRec MGNSKSGALSKEILEELQLNTKFTEEELSSWYQSFLKECPSGRITRQEFQTIYSKFFPEA

zRec2a MGNAKSSAMSKEILEDLKLNTKFSENELSQWYENFQKQCPSGRITPEEFKKIYERFFPEC

zRec2b MGNAQSGGIPREILDDLKLTTRFSESEITQWYENFQKQCPTGRITLQQFEEIYGKFFPDS

***::*..:.:::*::*: .* :::.:: ** .* ::**:***: ::*: ** ***:.

zRec1a DPTAYARHVFRSFDTNADGTLDFKEYIVALHLTSSGKTLRKLEWAFALYDVDGNGTISKN

zRec1b DPGAYAQHVFRSFDADSDGTLDFKEYIVALHLTSSGKTVEKLEWAFALYDVDRNGSITKN

bRec DPKAYAQHVFRSFDANSDGTLDFKEYVIALHMTSAGKTNQKLEWAFSLYDVDGNGTISKN

zRec2a DTTSYAQHVFRSFDTNDDGTLDFKEYIIALHMTSTGKTERKLEWAFSLFDVDKNGYITKS

zRec2b DATTYAQHVFRSFDANDDGTLDFKEYVVALHMTSSGKSTLKLEWAFSLFDVDKNGYVTKP

*. :**:*******:: *********::***:**:**: ******:*:*** ** ::*

zRec1a EVQEIVRSIFNMVPVEDQKNLPDDENTPEKRADKIWAFFGKQDNDKIGEGEFIQGVMENK

zRec1b EIHEIVKSIFNMISKEDQKNLPDDENTPEKRTDKIWDFFGKKENGKITEGEFIQGVMDNK

bRec EVLEIVTAIFKMISPEDTKHLPEDENTPEKRAEKIWGFFGKKDDDKLTEKEFIEGTLANK

zRec2a EVAEICQAIFKLIPKEDQESLPADENTPEKRADKLWSYFNKKDNERLAEGEFIQGILENE

zRec2b EVIELSQAIFKLIPKDKQTSLPNDESTPEKRAEKLWAIFDKKDNERVAEGEFIEAIQSSD

*: *: :**:::. :. ** **.*****::*:* *.*::: :: * ***:. ..

zRec1a DILRLIQYDEPKKIQEKLKEKKH

zRec1b HILRLIQFDKPQKVQEKLKEKTQ

bRec EILRLIQFE-PQKVKEKLKEKKL

zRec2a DAIHLIQFEHQKK----------

zRec2b DGLRLIQYDHK------------

. ::***::

**Figure S2:** Sequence alignment of bovine and zebrafish recoverin forms. Straight lines above the alignment indicate functional EF hands 2 and 3; dotted lines indicate non-functional EF hands 1 and 4. Several regions are highlighted with background colour: turquoise, hydrophobic amino acids of the myristoyl pocket (see Figure 6B); green, stretches of significant differences between bRec and zRec forms partially shown on Figure S3. Accession numbers are: NM_001030248.1 (zRec1a), NM_001317755.1 (zRec1b), NM_199964.1 (zRec2a), BC060902.1 (zRec2b).

Multiple sequence alignment was created with CLUSTAL W (1.81) (Thompson et al., 1994). The consensus is indicated as follows.

* - single, fully conserved residue

: - conservation of strong groups

. - conservation of weak groups

- no consensus

**References**

Thompson, J.D., Higgins, D.G. and Gibson, T.J. (1994) CLUSTAL W: improving the sensitivity of progressive multiple sequence alignment through sequence weighting, position-specific gap penalties and weight matrix choice. *Nucleic Acids Res*. 22, 4673–

4680

**Figure S3**

**
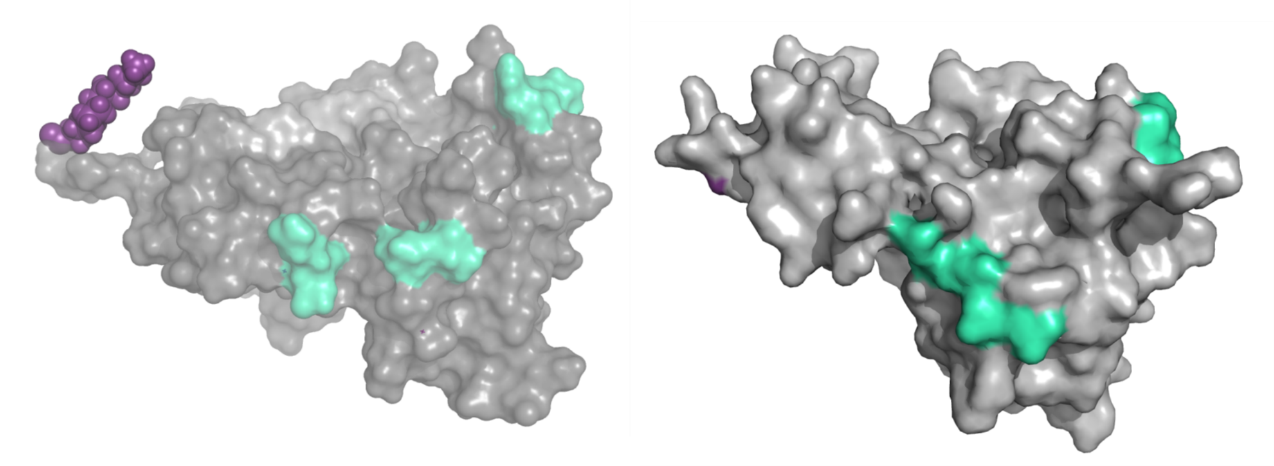
**

**Figure S3:** Small stretches of amino acid sequences that differ among bRec and zRec isoforms are highlighted in green. Images were created with Pymol; PDB code for myristoylated bRec was 1JSA, for non-myristoylated bRec was 1OMR.

**Figure S4**


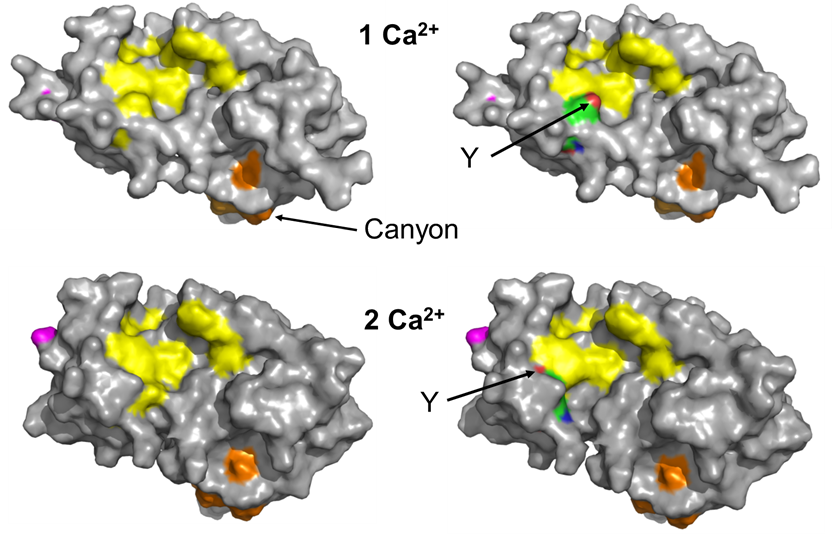


**Figure S4:** Comparison of three-dimensional structures of bovine recoverin with one and two Ca^2+^ bound (PDB codes 1OMR and 4YI8, respectively). Hydrophobic patches are labelled in yellow and orange. Phe23 (F) was replaced *in silico* by Tyr (Y) to illustrate a putative effect on the hydrophobic patch. Pink, position of G, where the myristoyl group would be attached.
